# Mapping Lower-Limb Prosthesis Load Distributions Using a Low-Cost Pressure Measurement System

**DOI:** 10.3389/fmedt.2022.908002

**Published:** 2022-06-17

**Authors:** Matthew O. Hopkins, Shruti Turner, Ravi Vaidyanathan, Alison McGregor

**Affiliations:** ^1^Biodynamics Laboratory, Department of Surgery and Cancer, Faculty of Medicine, Imperial College London, London, United Kingdom; ^2^Biomechatronics Laboratory, Department of Mechanical Engineering, Faculty of Engineering, Imperial College London, London, United Kingdom

**Keywords:** socket fit, pressure, sensors, IMU, lower-limb prosthesis, instrumentation, wearable

## Abstract

**Background:**

In the UK 55,000 people live with a major limb amputation. The prosthetic socket is problematic for users in relation to comfort and acceptance of the prosthesis; and is associated with the development of cysts and sores.

**Objectives:**

We have developed a prototype low-cost system combining low-profile pressure sensitive sensors with an inertial measurement unit to assess loading distribution within prosthetic sockets. The objective of this study was to determine the ability of this prototype to assess in-socket loading profiles of a person with an amputation during walking, with a view to understanding socket design and fit.

**Methods:**

The device was evaluated on four transtibial participants of various age and activity levels. The pressure sensors were embedded in the subject's sockets and an inertial measurement unit was attached to the posterior side of the socket. Measurements were taken during level walking in a gait lab.

**Results:**

The sensors were able to dynamically collect data, informing loading profiles within the socket which were in line with expected distributions for patellar-tendon-bearing and total-surface-bearing sockets. The patellar tendon bearing subject displayed loading predominately at the patellar tendon, tibial and lateral gastrocnemius regions. The total-surface bearing subjects indicated even load distribution throughout the socket except in one participant who presented with a large socket-foot misalignment.

**Conclusions:**

The sensors provided objective data showing the pressure distributions inside the prosthetic socket. The sensors were able to measure the pressure in the socket with sufficient accuracy to distinguish pressure regions that matched expected loading patterns. The information may be useful to aid fitting of complex residual limbs and for those with reduced sensation in their residual limb, alongside the subjective feedback from prosthesis users.

## Introduction

Major limb amputation affects up to five-thousand people a year in England alone (1, 2) and may occur as the result of dysvascular disease or from traumatic injury. The successful rehabilitation of individuals with prosthetic limbs contributes to a higher quality of life experienced for those with amputations (3). The prosthetic socket is frequently cited as the most important component of a lower- limb prosthesis with respect to user comfort and acceptance of the prosthetic limb, and successful rehabilitation (2, 4, 5).

The socket must enable load transmission and provision of good stability and control for locomotion (6), without causing discomfort or pain (7). Careful consideration is required from the prosthetist to ensure load is distributed effectively across tolerant regions for highly variable stump shapes and sizes (2). The interface pressure of the socket is affected by factors such as socket and residuum shape (6), socket alignment, suspension, residual limb site, and the ambulation task (e.g., level, inclined, uneven surfaces) (6). Lower-limb socket design has evolved since the generic prescription in the early 1900s. For transtibial amputations, the patella tendon bearing (PTB) socket design guides load to the patella tendon, an area deemed to be highly load-tolerant (8). In contrast, the total surface bearing (TSB) socket design aims to distribute the load evenly around the entire socket-residual limb interface to minimise the maximum pressure experienced across the residual limb (9).

The soft tissue of the residual limb is subjected to high epidermal stresses, shear stresses, and abrasive motions resultant from socket load transfer during ambulation (6, 7). These factors lead to intermittent skin deformation and tissue abrasion, generating heat, and moisture (6). Consequently, many individuals with amputations develop skin problems such as pressure sores, cysts, blisters, dermatitis, and oedema from using their prosthesis (6). Being able to quantify aspects of these stressors, particularly pressure may improve the design of sockets and our understanding of how to prevent the associated complications. It has been reported that up to a quarter of prosthesis users avoid regular use of their artificial limb, citing discomfort, pain, and poor fit as the cause (2, 6).

Prosthetists attempt to control interface and shear stress combinations through alteration of socket shape, liner material, alignment, and prosthesis componentry using the subjective feedback from prosthesis users (6, 10). However, the definition of a “good socket fit” is vague (2) and fitting is highly dependent on the skill and experience of the prosthetist (2). Acceptable levels and combinations of normal and shear stress are not well understood and may limit the quality of socket fit, potentially contributing to the frequency of skin breakdown and infections experienced by prosthesis users (7, 10). The issues are exacerbated for individuals who cannot give reliable feedback about the pressure felt on their residual limbs e.g., due to nerve damage, skin grafting. The use of sensors in the socket would allow for objective pressure measurements to be used in conjunction with the subjective feedback from prosthesis users to guide prosthetists with prosthetic fitting.

It is crucial to develop a better understanding of the biomechanical coupling between the prosthetic socket and the residual limb in order to improve socket fit (6). Previous literature has shown the ability of researchers to create sensing technologies for potential use in prosthetic applications (11). However, the majority of these sensors have not been created with the involvement of the clinicians and users, the people who will use the technology (11). Many of the sensors created are bulky or require wired connection to a computer and specialist knowledge to use (11), none of which are appropriate for the clinical environment. A successful tool would benefit from being low-cost to manufacture, using relatively simple methods, have a minimal thickness, be flexible to shape around the curves of the socket-residual limb interface, and demand minimal training and operating time to effectively fit in with clinical requirements (2).

**Figure 1:**
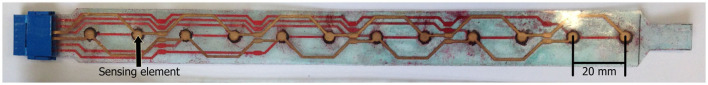
Low-cost, flexible pressure sensor strip containing 12 sensing elements.

A smart-socket system has been developed as a low-cost, low-profile device for use as a feedback tool indicating within socket loading profiles to aid in the fitting phase of socket production and subsequent rehabilitation (12). The smart-socket is comprised of a socket with 12 strips of 12 piezoresistive sensors (144 sensors in total), covering the inner surface. The sensors were produced using a low-cost piezoresistive material known as Velostat, chemical etched copper electrodes and an acetate backing. Solid ink printing was used to produce electrode patterns on copper sheets in top and bottom layer configurations. These were then backed with an adhesive sheet and acetate, before undergoing chemical etching to remove unwanted copper. Once etched, the bottom layer was fitted with another adhesive layer with holes punched in it to enable Velostat element placement. Punched Velostat discs were placed into each hole and the upper electrode layer was then attached to complete the sandwich structure. The sensors were constructed in a sandwich strip format (see Figure 1) providing twelve individual sensing elements in a flexible, low-profile arrangement. The aim was for total coverage, with a gap of ~2 cm between sensors in all directions. The sensors are ~1 mm thick and are made of flexible plastic for durability and easy molding around the irregular surface shape of the inner socket.

Data is sampled from the sensors at a rate of 200 Hz and transmit it to a smartphone application. Prior work examined the properties of these sensors under bench testing conditions, yielding cyclic drift measurements of up to 0.00715V/cycle (0–3.3V supply), a static drift of up to 1.17%/min of the full-scale output (FSO) and a hysteresis effect of up to 7.25% FSO (12). Thermal drift tests indicated minor fluctuations in response, but no discernable pattern (12). The sensors typically displayed a working resistance range between 33.33 MΩ and 5.72 kΩ with an initial sharp decrease in resistance, followed by a linear region between 100kPa and 400 kPa (12). Despite accuracy errors in the range of 16 to 48% of the full-scale range, the sensors demonstrated that they were able to reflect expected loading patterns within an ischial containment socket (12).

Prior work demonstrated the device could provide relevant and important clinical information; however, improvements are required to obtain an output as a real metric (12). The purpose of this study was to capture lower-limb prosthetic socket loading profiles using the sensors during standing and walking, and compare the pressure maps formed with expected load distributions, based on the socket type, to determine their suitability in identification of pressure distribution within the socket.

## Materials and Methods

A preliminary cohort study was conducted on a group of subjects with lower-limb amputations that were asked to walk across the gait laboratory at a self-selected speed. Ethical approval was secured for the trial (London Riverside Research Ethics Committee, REC reference: **15/LO/1633**, IRAS project ID: **177122**).

A total of four transtibial participants were recruited from our local limb fitting centre with a mixture of amputation types (Table 1), and informed written consent obtained. Three participants were fitted with total-surface bearing sockets, and one was fitted with a patellar-tendon bearing socket. The inclusion and exclusion criteria for the study are documented in Table 2.

**Table 1:** Participant Information.

**Subject**	**S001**	**S002**	**S003**	**S004**
Amputation type.	Unilateral transtibial.	Unilateral transtibial.	Unilateral transtibial.	Unilateral transtibial.
Amputated leg.	Left.	Right.	Left.	Right.
Instrumented leg.	Left.	Right.	Left.	Right.
Age (years)	74	24	50	47
Sex	Male	Female.	Female.	Male.
Height (cm)	177.2	164.0	172.7	181.5
Weight (with prosthesis) (kg)	78.2	90.0	114.6	104.6
Weight of the prosthesis (kg)	2	2.2	2.6	2.8
Self-reported fitness level	3/5	2/5	2/5	3/5
Length of rehabilitation	12+ months.	12+ months.	3 months.	12+ months.
Known gait deficiencies in addition to the amputation.	None.	None.	Back cramping due to posture.	None.
Number of smart-socket strips used.	11	12	10	10
Additional information.	–	Foot aligned laterally.	–	–
Socket Type.	PTB.	TSB.	TSB.	TSB.
Pin Lock.	Yes	No.	Yes.	Yes.
Liner Type.	Pe-Lite.	Alps cushion.	Endolite.	Pe-Lite.
Ankle.	Multiflex.	Echelon.	Espirit.	Multiflex.

**Table 2:** Inclusion and exclusion criteria for participation in the study.

**Inclusion Criteria**	**Exclusion Criteria**
Aged between 18 and 85.	Volunteers who are not confident in use of their prosthetic limb. Volunteers who do not speak fluent English.
Must have achieved confidence in use of their prosthesis.	Persons whose cognitive function will prevent them from understanding the study.
Free of any medication or condition that may influence the ability to move freely.	Persons with any musculoskeletal injury, impairment or co-morbidity that may impair their ability to function normally.
Good understanding of written and spoken English.	Any current stump conditions e.g., pressure sore, wound breakdown.

**Figure 2:**
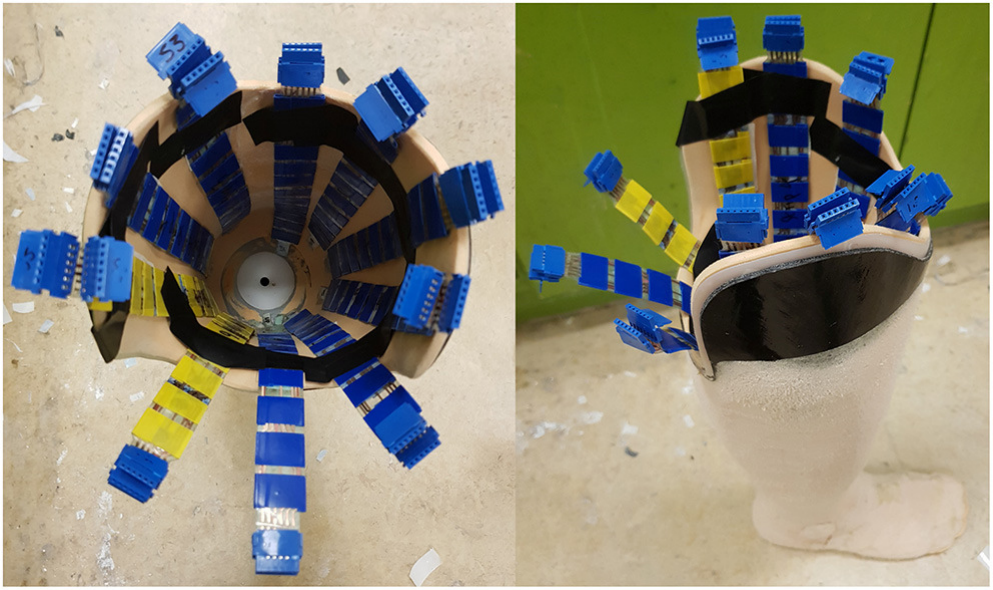
Interior and exterior of a socket in which sensors have been fixed with double-sided adhesive tape.

**Figure 3:**
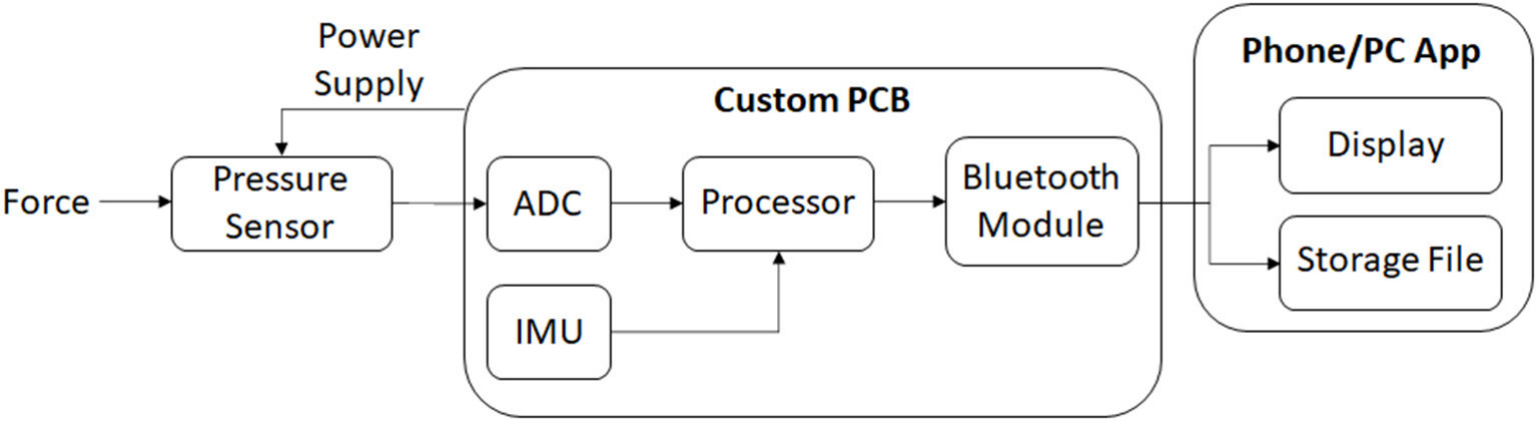
Flow diagram indicating the data collection process.

The developed pressure sensors were placed into each subject's socket and secured with double sided adhesive tape (see Figure 2). Ten or more sensor strips, each comprised of 12 individual sensors, were placed in each socket depending on the size of the socket. Strips were evenly distributed within the socket with a focus on regions of interest, such as the bony landmarks of the residual limb. In some instances, sensors protruded from the socket, in particular at the posterior-proximal region. A bespoke inertial measurement unit (IMU) with triple-axis gyroscope, magnetometer, and accelerometer was attached to the posterior side of the socket using Velcro. Sensor strips were wired directly into this IMU, supplied by the 3.3 V rail and sampled *via* a microcontroller analogue-to-digital converter (ADC) with a 10-bit resolution. Data was sampled at a rate of 200 Hz and transmitted *via* Bluetooth to a nearby computer system (see Figure 3). Participant motion was captured using a Vicon T020 motion capture suite utilising a modified Helen Hayes (MHH) marker set to capture hip and lower limb motion (13). Motion capture data was used to verify phases of the gait cycle as detected by the IMU, but was not included in the analysis.

Static stance measurements were taken whilst the participant stood with feet together and arms spanned in a T-pose, walking measurements were taken after the static stance. During the walking task, subjects traversed the laboratory at a self-selected speed back and forth five times. From this data, five central steps, in which the participant passed over the central portion of the walkway, and five turning steps were extracted. The selection ensured a stable step unperturbed by turning or changes in acceleration associated with beginning or terminating a new walk.

Data from the smart socket was analysed offline using MATLAB, alongside the Vicon data that was used to verify the smart socket analyses. The pressure and inertial data were segmented into gait cycle phases based on acceleration and gyroscope peaks corresponding to heel-strike and mid-swing, respectively. Vicon data was synchronised using the observed motion capture heel-strike frame. Sensor response was normalised on a per sensor basis across all activities (see Equation 1), producing an output that reflected a sensor's instantaneous output in relation to its maximum and minimum observed pressures throughout the trial. All measurements were normalised to be relative to the T-Stance data taken at the beginning of each participant study.


(1) $$\begin{array}{r} x_{n}=\frac{x-\;x_{min}}{x_{max}-x_{min}} \end{array}$$

Where *x*_*n*_ is the normalised data point, x is the original reading, *x*_*max*_ is the maximum observed sensor reading and *x*_*min*_ is the minimum observed sensor reading.

Loading profile maps were generated from the normalised sensor output. Each segment of the maps corresponds to a sensor, with the inner-most sections representing the distal-most sensors, and top and bottom positions on the maps aligning with anterior and posterior sensors, respectively. A pressure map was generated for the standing task from the mean value of each sensor during this task (nominally 2–3 seconds). Similarly, data was averaged across five instances of heel-strike and mid-swing during central walking steps and turning steps as observed in the motion capture data. In some instances, connections to sensors were damaged, preventing readings. Affected sensors have been marked in the maps by cross-hatching.

We calculated a centre of pressure metric, referred to as the locus of maximum pressure (LMP), from the normalised outputs for each sensor (see Equation 2), reflecting the highest concentration of maximal sensor output. The pressure distributions and movement of the LMP were extracted for points of interest such as loading of the limb during stance-phase. Position of the locus of maximum pressure and the average load values were compared with visual interpretation of the motion tracking data.


(2) $$\begin{array}{r} x_{LMP}=\frac{\int xp\left(x\right)dx}{\int p\left(x\right)dx} \end{array}$$

Where *x*_*LMP*_ is the coordinate of the LMP in a single axis, x is the position of the centroid of the sensor representation from the centre of the socket representation (that being the distal-most end) and *p*(*x*) is the pressure reading from the sensor.

## Results

Pressure profiles were generated using normalised sensor data for each participant during standing, walking stance-phase and walking swing-phase. During the walking task, both central steps and turning steps were extracted in which the participant was at the centre of the walkway and at the edge of the walkway, respectively. These allowed observation of unperturbed walking and directional change. Pressure maps were generated using data averaged across the duration of the standing task and across five central gait cycles and five turning gait cycles, respectively.

**Figure 4:**
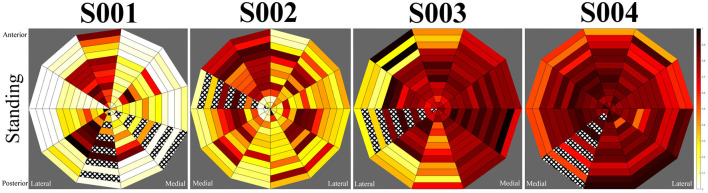
Mean standing pressures during the static for participants. Left to right, subjects S001 to S004.

Pressure profiles were produced for each participant whilst standing still (see Figure 4). In the patellar-tendon-bearing (PTB) case (subject S001) a distinct pattern emerged within the pressure map, indicating primary loading of the residual limb at the patellar tendon, tibial region, and lateral gastrocnemius muscle regions. The remaining subjects utilised a total-surface-bearing (TSB) socket. During standing subjects S003 and S004 data indicated an even pressure distribution across their sockets. Subject S002 produced a standing pressure profile like the patellar tendon bearing case with loading appearing concentrated about the tibial, patellar, distal-medial, and distal-lateral regions. The corresponding pressure maps for each subject are displayed in Figure 4.

**Figure 5:**
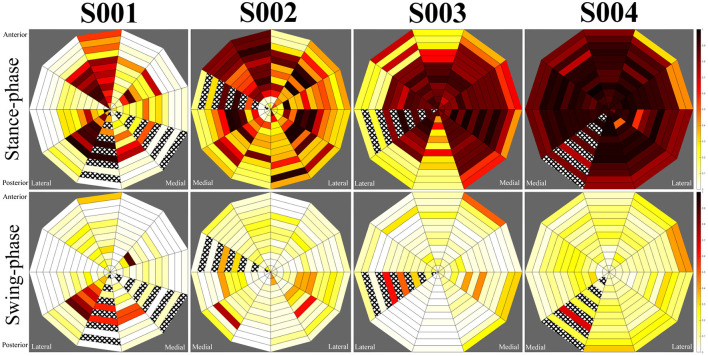
Average central step pressure maps for each subject. Top row: stance phase, bottom row: swing-phase.

**Figure 6:**
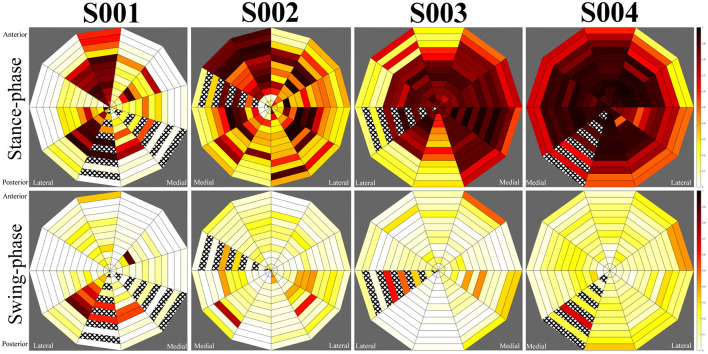
Average turning step pressure maps for each subject. Top row: stance phase, bottom row: swing-phase.

During walking stance-phase, similar patterns were observed for each subject in comparison to their standing loading profiles, however, the magnitude of loading was greater (see Figure 5). Variations were indicated in the loading regions for several subjects depending on whether they were walking in the centre of the walkway or performing a turning motion at the walkway's extremes. These variations were most prominent for subjects S003 and S004 (see Figure 6), whilst minimal differences were observed between central and turning steps for the first two subjects, suggesting the load is borne in distinct areas of the socket regardless of variation in common walking motions. Subject S003 displayed greater load variation on the mid-medial side of the socket, whilst subject S004 displayed lower loading of the proximal rim during left-turns and greater loading in the proximal rim during right-turns. The observed fluctuations appeared to align with a wider dynamic stance when this subject was turning, causing the prosthesis to angle more than with other participants.

During swing-phase, loads across the socket were significantly reduced for each participant. Small loads were indicated in select regions for each individual. In the patellar-tendon bearing case (subject S001), loading remained at the lateral distal region of the socket, corresponding to the gastrocnemius. Subjects S002 and S003 displayed moderate loads at the distal medial and distal lateral regions, corresponding to the lower calf muscle region. Subject S004 indicated a greater level of contact between the socket and residuum during swing-phase, however, this remained evenly distributed across the socket. Swing-phase patterns remained largely consistent for each participant regardless of whether they were turning.

A pseudo centre of pressure measurement, named the locus of maximum pressure (LMP), was determined for each participant. This value reflects the point within the socket at which the greatest concentration of sensors displayed their highest outputs. The movement of this value across the socket provides an indication of the transfer of load across the socket during dynamic walking motion.

For subject S001, using a patellar-tendon bearing socket, observation of the LMP indicated a linear movement between the posterior and anterior aspects of the limb from swing-phase to stance-phase, respectively. The LMP in this instance had an offset towards the mid-lateral side of the socket. For subject S002, the LMP moved from posterior-lateral position to anterior-medial when loading. The pressure map indicated unloading of the limb in a clockwise manner. For S003, the LMP moved from the anterior position to medial position during loading. During unloading, elevated pressure occurred at the proximal posterior position, pulling the LMP posteriorly. S004 displayed greater motion in the medial and lateral directions during walking than other participants.

## Discussion

The aim of this study was to determine whether the sensor network had the potential for clinical benefit through collection of pressure distribution data in the socket. Data collected from the smart-socket device for the participants indicated the system was able to provide a measure of contact distribution between the residual limb and the socket in addition to a relative measure of load. Given the capabilities of the smart socket confirmed in this study, the set up may be appropriate for use in the clinical environment to aid prosthetic socket fitting.

The data collected by the system consistently reflected the gait characteristics recorded by motion tracking. The patellar-tendon bearing (PTB) socket was expected to produce more distinct loading profiles than total-surface bearing (TSB) sockets, with particular load concentration about the patellar tendon, the medial tibial flare and the lower portion of the tibial medial condyle (7, 14). Loading was also expected at the anteromedial and anterolateral tibia, the shaft of the fibula, and at the posterior compartments of the leg (7).

Measurement of subject S001, using a PTB socket, displayed prominent loads about the patellar-tendon, tibial, and lateral gastrocnemius regions. These observations agreed with the expected load pattern (8) and suggested a successful targeting of the patella tendon region by the prosthetist in production of this subject's prosthesis. Observation of the LMP indicated a linear movement between the posterior and anterior aspects of the limb from swing-phase to stance-phase, respectively with an emphasis on loading of the lateral side of the limb. This implied minimal twisting of the prosthetic limb, which is beneficial for prosthesis control and cause issues with the residual limb due to friction and associated shear (15).

The remaining three subjects used TSB sockets in which the load distribution is expected to be even across the entirety of the socket (9). An even loading pattern across the socket was present for subjects S003 and S004, however, subject S002 displayed a pattern similar to that observed in the PTB socket. Subject S002 utilised an unusual prosthetic alignment in which the foot was displaced laterally. The alignment of the prosthesis may be responsible for the imbalance in loading observed in this socket. Load was distributed toward the medial side of the tibia and the mid-lateral gastrocnemius regions. Additionally, the LMP moved from posterior-lateral position to anterior-medial when loading, suggestive of load rotating around the socket. Whilst the user was able to perform the required activities of the study, there may be long-term health implications of a laterally aligned prosthesis due to the unexpected pressure distribution in the socket, such as residual limb issues and musculoskeletal overuse injuries (16). Due to the lack of documentation of clinical decision making when fitting prosthetic sockets and prostheses (17), it is not possible to know why the prosthesis was set up in this way other than for user preference. Further analysis of the movement data would help to understand the impact on the participant's motion, thus predicting the likelihood of longer-term injury.

Minor variations in the loading patterns were observed for subjects S003 and S004 when turning. For subject S003 greater load variations were visible on the mid-medial side of their socket, possibly suggestive of a looseness of fit. Elevated pressure at the proximal posterior region of the socket during unloading of the prosthesis may indicate pistoning, as other participants tended to experience a reduction of load at positions of the socket that were already loaded. Subject S004 experienced lower pressures across the proximal rim of the socket when turning left, suggestive of an angling of the residual limb. This observation was confirmed by a wide stance when turning in the motion capture data.

It is difficult to compare the results to those of other studies due to their different focus. Whilst other studies do measure the pressure inside the prosthetic socket, the focus has been on maximum pressure readings at specific locations of interest, rather than an overall understanding of the pressure distribution. Commercial technologies do exist to measure pressure in the socket (18, 19), but none found provide pressure measurements across the entire inner surface of the socket, rather focusing on particular regions. The Adapttech Insight system is the closest system to the one tested in this study (20), however, from the information they have published it is not usual to use so many sensors in the socket (21). All the commercial options are associated with high costs and some require wired connection to a computer, yielding them inaccessible to moth healthcare settings.

The results indicate that the sensor network can map contact distribution throughout the socket in real-time, providing indications of how loading is transferring across the residuum during motion. This may useful to clinicians when fitting sockets, supplying them with information such as the closeness of fit, change in fit over time and motion of the socket in relation to the residuum. By providing direct feedback on these factors, prosthetists will have more information from which to estimate the effects of their design choices on skin and soft tissue health. The next steps of this work are to refine the sensing hardware and software interface to refine the information and usability of the system.

The smart socket has not yet been tested in the clinical environment with clinicians as, due to their strained time, it was deemed appropriate to provide evidence of their value before taking up clinical time. This study is an initial step to demonstrate the functionality and value of the smart socket, which can then be enhanced for testing in the clinical environment.

The system was not able to produce accurate measures of absolute pressure, due to the limitations of the transducer. However, the objective measurement of the socket's relative pressure distribution will allow clinicians to “see” whether their socket design and prosthesis set up is as intended relative to the socket types. The LMP gives an indication of the residual limb movement within the socket, which may not always be reported or noticed, particularly if it is not vertical i.e., pistoning. The change in LMP may indicate the movement of internal structures, e.g., bone, that may cause damage over time which may not be noticeable if the residual limb does not move in its entirety.

Before the smart sockets are incorporated into clinical practice, further engagement with clinicians and users is vital to determine how the tool can be used and if they are willing to accept them into routine practice. Involvement is also important to design the software which must be used to receive and display the information.

The results from this study show the clinical benefit the smart sockets could have, with each participant's results indicating that the system is capable of reflecting socket loading distributions. This information may be of use to prosthetists when attempting to fit sockets as it could provide a clearer and less skill-dependent indication of the overall loading profile than traditional techniques such as clear check sockets and skin blanching (14). Improvements are being made to the sensors to improve accuracy and repeatability, along with further testing to confirm initial results. The low-cost nature of this device lends itself to clinical adoption, a common barrier to integration of existing solutions (2).

In conclusion, the sockets of four participants with transtibial amputations were instrumented with low-cost, low-profile sensors. Initial studies demonstrate the sensors can produce useful indications of residuum-socket contact and load distribution inside the socket. The information collected by the sensors shows their potential clinical utility in aiding prosthetists with socket fitting, by providing quantitative information to complement their skills and the subjective feedback from prosthesis users. Work is ongoing with prosthetists and physiotherapists to further develop the hardware and understand how they would like to use the information *via* appropriate visualisation methods.

## Data Availability Statement

The datasets presented in this article are not readily available because Ethics restrictions mean the data cannot be shared. Requests to access the datasets should be directed to a.mcgregor@imperial.ac.uk.

## Ethics Statement

The studies involving human participants were reviewed and approved by the Integrated Research Application System, Imperial College London. The patients/participants provided their written informed consent to participate in this study.

## Author Contributions

MH led the writing of the manuscript. All authors contributed equally through critical review and editing of the manuscript. All authors contributed to the article and approved the submitted version.

## Funding

This work was conducted under the auspices of the Centre for Blast Injury Studies at Imperial College London. The authors would like to acknowledge the financial support of the Centre for Blast Injury Studies.

## Conflict of Interest

The authors declare that the research was conducted in the absence of any commercial or financial relationships that could be construed as a potential conflict of interest.

## Publisher's Note

All claims expressed in this article are solely those of the authors and do not necessarily represent those of their affiliated organizations, or those of the publisher, the editors and the reviewers. Any product that may be evaluated in this article, or claim that may be made by its manufacturer, is not guaranteed or endorsed by the publisher.
